# Bacteria-induced egg hatching differs for *Trichuris muris* and *Trichuris suis*

**DOI:** 10.1186/s13071-015-0986-z

**Published:** 2015-07-15

**Authors:** Nermina Vejzagić, Roberto Adelfio, Jennifer Keiser, Helene Kringel, Stig Milan Thamsborg, Christian M.O. Kapel

**Affiliations:** Section for Organismal Biology, Department of Plant and Environmental Sciences, Faculty of Science, University of Copenhagen, Frederiksberg C, Denmark; Medical Parasitology and Infection Biology, Swiss Tropical and Public Health Institute, Basel, Switzerland; University of Basel, Basel, Switzerland; Parasite Technologies A/S, Hørsholm, Denmark; Parasitology and Aquatic Diseases, Department of Veterinary Disease Biology, Faculty of Health and Medical Sciences, University of Copenhagen, Frederiksberg C, Denmark

**Keywords:** *Trichuris muris*, *Trichuris suis*, *In vitro*, Egg hatching, Bacteria

## Abstract

**Background:**

Eggs of the porcine whipworm *Trichuris suis* are currently explored in human clinical trials as a treatment of immune-mediated diseases. In this context, only the infective, embryonated eggs, constitute the Active Pharmaceutical Ingredient (API). The rodent whipworm, *Trichuris muris* is commonly used as a laboratory model to study *Trichuris* biology. The embryonated eggs (containing a fully developed larva) are biologically active and will invade the large intestinal mucosa of the host. This study aims to assess the *in vitro* hatching of *T. muris* and *T. suis* eggs in various bacterial cultures as a measure for their biological activity.

**Methods:**

Eggs of *T. muris* and *T. suis* were incubated with *Escherichia coli* strain (BL-21) at three concentrations in a slightly modified *in vitro* egg hatching assay previously developed for *T. muris*. Additionally, *E. coli* strains (M15, SG13009, PMC103, JM109, TUNER, DH5alpha, TOP10) and five Gram-positive bacteria (*Enterococcus caccae*, *Streptococcus hyointestinalis*, *Lactobacillus amylovorus*, *L. murinus,* and *L. reuteri*) were tested as a hatching stimulus for *T. muris* and *T. suis* eggs.

**Results:**

Whereas *T. muris* eggs hatched, *T. suis* did not, even when exposed to different concentrations and strains of *E. coli* after 4 and 24-hour incubation. When incubated with Gram-positive bacteria, only *T. muris* eggs showed noticeable hatching after 20 h, although with high variability.

**Conclusions:**

The observed difference in hatching of *T. muris* and *T. suis* eggs incubated with selected bacteria, indicate significant biological differences which may reflect specific adaptation to different host-specific gut microbiota.

## Background

*Trichuris muris* and *T. suis,* whipworms of mice and pigs, respectively, invade the mucosa of the large intestine [1–3]. The life cycle is direct with embryonated eggs (each containing a fully developed larva) as infective stage [1, 3]. The embryonated eggs of *T. suis* (TSO) represent a raw material of the Active Pharmaceutical Ingredient (API) in a medicinal product, which is currently being explored as a treatment for patients with immune-mediated diseases. Such helminthic therapy is founded on the hygiene hypothesis [4] or more broadly, the ‘Old friends’ hypothesis [5, 6]. In more details, a positive immunomodulatory effect of TSO has been demonstrated in patients with inflammatory bowel disease [7–9] and multiple sclerosis [10], although this positive immunomodulatory effect was not demonstrated in a safety and efficacy study in patients with multiple sclerosis [11]. The treatment with TSO is well-tolerated in patients and only transient gastrointestinal symptoms are present following first dosing [10–15].

A first requirement to use TSO as a medicinal drug is that the eggs are biologically active, i.e. that the eggs are able to hatch for the release of larvae. Description of eggs based solely on their morphological development and appearance of larvae inside does not necessarily correlate with the egg hatchability, as larvae may degenerate over an extended period of storage.

In recent years, individual bacterial species were successfully used for hatching *T. muris* eggs under *in vitro* conditions [16–18]. However, whether *T. suis* eggs share the same egg hatching stimulation as *T. muris* eggs is still unknown. Therefore, in the present study a slight modification of a published method based on Gram-negative *Escherichia coli* (BL-21) for hatching *T. muris* eggs [16] was employed to test whether *T. muris* and *T. suis* eggs show different ability to hatch when incubated under the same conditions. Further, eggs of the two *Trichuris* species were incubated with five Gram-positive bacteria.

Here we report how two very closely related nematodes (same genus) respond differently to *in vitro* hatching when incubated with selected Gram-negative and Gram-positive bacteria.

## Methods

### Parasite batches

Eggs of *T. muris* were isolated from faeces of experimentally infected female mice (C57BL/10ScSnOlaHsd) and were embryonated for 3 months at room temperature (23–24 °C) in Milli-Q (ultrapure water purified by filtration and deionization) and in sulphuric acid (H_2_SO_4_) pH1, respectively.

Eggs of *T. suis* were isolated from faeces of experimentally infected female Göttingen minipigs (Ellegaard Göttingen Minipigs A/S, Dalmose, DK). The eggs were embryonated in H_2_SO_4_ pH1 for 100 days (±10 days) at 25 °C and then stored at 5 °C (2–8 °C).

Stabilized H_2_SO_4_ pH1 was prepared by adding 94 ml 1 M H_2_SO_4,_ 36 mg potassium sulphate and 1236 mg sodium sulphate in 1000 ml distilled water to prevent any bacterial and fungal growth during storage of *T. suis* eggs. Storage in sulphuric acid is not known to affect either the development or the infectivity of *T. suis* eggs [19]. Also, 0.1 N H_2_SO_4_ has been used as an embryonation medium for *Ascaris suum* eggs [20].

To secure comparable preparatory conditions for the batches in the experiment with Gram-positive bacteria, the medium of one *T. muris* batch was changed to H_2_SO_4_ pH1 and stored at 5 °C, and the medium of one *T. suis* batch was changed to Milli-Q and stored at room temperature for 3 months.

Prior to use, all batches were transferred to Milli-Q.

### Bacteria

The *E. coli* strains (BL-21, M15, SG13009, PMC103, JM109, TUNER, DH5alpha, TOP10) were incubated overnight in Luria Broth (Sigma-Aldrich) at 37 °C on a shaker (250 rounds per minute; rpm). The Gram-positive bacteria *Streptococcus hyointestinalis* (DSM No. 20770) and *Enterococcus caccae* (DSM No. 19114) were cultured in tryptic soy broth (Sigma-Aldrich) with added yeast extract (Sigma-Aldrich). *Lactobacillus amylovorus* (DSM No. 16698, deposited by Dr. S. Konstantinov, Laboratory for Microbiology, Wageningen University and Research Centre Agrotechnology & Food Sciences, Wageningen, The Netherlands), *Lactobacillus murinus* (DSM No. 20452), and *Lactobacillus reuteri* (DSM No. 20016) were cultured in MRS broth (Sigma-Aldrich). Cultures were incubated overnight in the presence of CO_2_ on a shaker at 37 °C (range 220–275 rpm).

The optical density (OD) was measured on SpectraMax M2 (Molecular Devices) at 600 nm (absorbance). The concentration was calculated using *E. coli* formula: OD × 5 × 10^8^ = bacteria/ml [16].

### Incubation with *Escherichia coli*

A total of 1000 embryonated eggs per well were plated on 24-well plates in RPMI (1640, W/HEPES; CE, GIBCO) with 5 % penicillin (Invitrogen, 100 U/ml) -streptomycin (Invitrogen, 100 μg/ml) and 5 % amphotericin B (Sigma-Aldrich A2942, 250 μg/ml) together with an *E. coli* suspension.The samples were incubated at 36 °C and examined after 4 and 24 hours. The BL-21 strain (*E. coli* strain used for hatching *T. muris* eggs as described in [16]) was tested at three concentrations (5×10^8^, 10×10^8^ and 20×10^8^ bacteria/ml), while the other *E. coli* strains were tested at one concentration (5×10^8^ bacteria/ml). After the incubation, the wells with *T. muris* eggs were examined for hatching in subsamples (4–5 × 20 μl), whereas entire wells were examined for *T. suis* eggs.

Furthermore, the *E. coli* hatching assay was performed on *T. muris* eggs embryonated in Milli-Q or H_2_SO_4_ pH1 in concentrations of 600–1500 embryonated eggs in 3 ml, according to the original method. The number of released larvae was examined in subsamples for n = 4 per group.

### Incubation with Gram-positive bacteria

A suspension of Gram-positive bacterium (5×10^8^ bacteria/ml) was mixed with *Trichuris* eggs, and five aliquots of approximately 100 embryonated eggs per well were plated on a 96-well plate. Plates were flushed with CO_2_ and incubated in RPMI-1640 alone or RPMI-1640 containing 5 % penicillin-streptomycin and 5 % amphotericin B at 37 °C. The samples were examined after 4 and 20 h.

In all experiments, released larvae were enumerated using an inverted microscope (Carl Zeiss Primo Vert).

### Statistical analysis

Data was analyzed in GraphPad Prism software (version 6) and R version 3.1.2 (R Core Team, 2014). Hatching percentage was calculated based on the number of released larvae and the number of embryonated eggs added per well. Data was log transformed as y = log (y + 1) whenever necessary to fulfill requirements of the normal distribution for the analysis. One-way ANOVA was used to test the effect of different *E. coli* (BL-21) concentrations on *T. muris* egg hatching after 4, and 24 h, followed by the Tukey’s *post hoc* test for multiple comparisons. The effects of different *E. coli* strains and the time of incubation on *T. muris* egg hatching were evaluated with the two-way ANOVA. Since the hatching in the control groups was absent or close to 0, they were not included in the analysis of ANOVA. An unpaired Student’s *t* test was used for pairwise comparisons. Generalized linear mixed model (glmm) fit by maximum likelihood (Laplace approximation) (Bates D, Maechler M, Bolker B and Walker S, 2014; R package version 1.1–7) was used to test the influence of different bacteria and the type of egg storage medium (Milli-Q and H_2_SO_4_ pH1) on overnight *T. muris* egg hatching in RPMI-1640 without antimicrobials. As data contained too many 0 values, the interaction between bacteria and the type of egg storage medium (Milli-Q and H_2_SO_4_ pH1) was not included in the analysis. Values of *p* < 0.05 were considered statistically significant.

### Ethical approval for animal use

The experimentation on mice was approved by the local veterinary agency based on Swiss cantonal and national regulations (permission no. 2070), and on minipigs by the Animal Experiments Inspectorate, Ministry of Food, Agriculture and Fisheries of Denmark (permission no: 2012-15-2934-00641).

## Results

### Egg hatching with *Escherichia coli*

After 4 h incubation, the two lowest concentrations of *E. coli* (BL-21) tested (5×10^8^ and 10×10^8^ bacteria/ml) resulted in significantly higher *T. muris* egg hatching than a concentration of 20×10^8^ bacteria/ml (F_2,9_ = 54.20, *p* < 0.0001; Tukey’s *post hoc* test *p* < 0.0001). However, no significant difference could be demonstrated after 24 h (F_2,9_ = 0.93, *p* = 0.43). No hatching was observed for *T. suis* at any concentration tested (Table 1).

**Table 1:** Hatching of *Trichuris muris* and *T. suis* eggs after incubation with *Escherichia coli* (BL-21)

	Average hatching percentage (± SD)			
*Escherichia coli* concentration/ml	4 h		24 h	
	*T. muris*	*T. suis*	*T. muris*	*T. suis*
5 × 10^8^	83.9 (±12.9)	0.1 (±0.1)	80.5 (±16.9)	0.1 (±0.1)
10 × 10^8^	57.0 (±1.4)	0.0 (±0.1)	70.5 (±4.8)	0.1 (±0.1)
20 × 10^8^	13.5 (±4.8)	0.0 (±0.0)	72.0 (±21.8)	0.0 (±0.0)
Controls (no *E. coli*)	0.0 (±0.0)	0.2 (±0.2)	0.1 (±0.1)	0.2 (±0.2)

*Trichuris* eggs incubated with three different concentrations of *E. coli* (BL-21) in RPMI-1640 with 5 % penicillin-streptomycin and 5 % amphotericin B after 4 and 24 h incubation at 36 °C. The controls are *Trichuris* eggs incubated without the *E. coli*; n = 4 per group

**Fig. 1:**
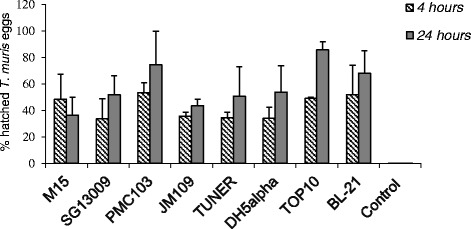
*Trichuris muris* eggs incubated with different *Escherichia coli* strains. Samples were incubated for 4 and 24 hours at 36 °C. The control group represents *T. muris* eggs incubated without the addition of *E. coli*. Arithmetic mean (bar) and standard deviation (error bars) for n = 3 per group

The strain of *E. coli* (ANOVA F_7, 32_ = 3.52, *p* = 0.01) and the time of incubation (ANOVA F_1, 32_ = 12.97, *p* = 0.001) had a significant effect on the hatching of *T. muris* eggs (Fig. 1). None of the *E. coli* strains evaluated induced hatching of *T. suis* eggs after 4 and 24 h incubation (average hatching was less than 0.2 %).

No significant difference in hatching was observed for *T. muris* eggs embryonated in H_2_SO_4_ pH1 (10.9 ± 6.3 %) (mean ± S.D.) and in Milli-Q (21.1 ± 8.4 %) after 4 h with *E. coli* at 37 °C (5×10^8^ bacteria/ml) (t_6_ = 1.9, *p* = 0.10).

### Egg hatching with Gram-positive bacteria

**Fig. 2:**
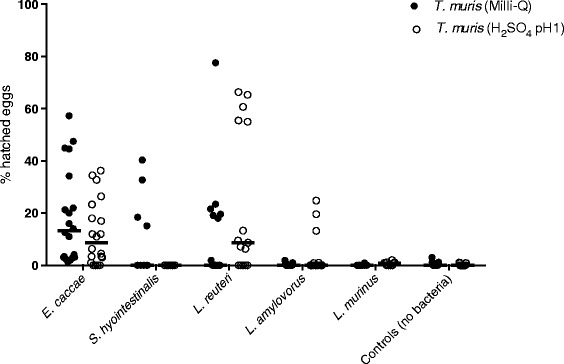
*Trichuris muris* eggs incubated with Gram-positive bacteria in RPMI-1640 without antimicrobials. Samples were incubated for 20 hours at 37 °C. Individual counts of *T. muris* (Milli-Q or H_2_SO_4_ pH1) and median for all experiments with *Enterococcus caccae* (n = 20), *Streptococcus hyointestinalis* (n = 10), *Lactobacillus reuteri* (n = 15), *L. amylovorus* (n = 15), and *L. murinus* (n = 10). Controls represent *T. muris* eggs incubated without the addition of bacteria (n = 25)

After 4 h, no significant hatching could be demonstrated for *T. muris* incubated with any of the Gram-positive bacteria in any incubation media (the highest average hatching was 0.6 %). After 20 h, *E. caccae* (z value = 4.26, *p* < 0.001) and *L. reuteri* (z value = 3.42, *p* < 0.001) induced significantly higher hatching of *T. muris* eggs as compared to the controls. The effect of *L. reuteri* on egg hatching did not significantly differ from *E. caccae* (*p* = 0.62) (Fig. 2). However, high variation was observed in and in-between experiments with the same bacterium.

**Fig. 3:**
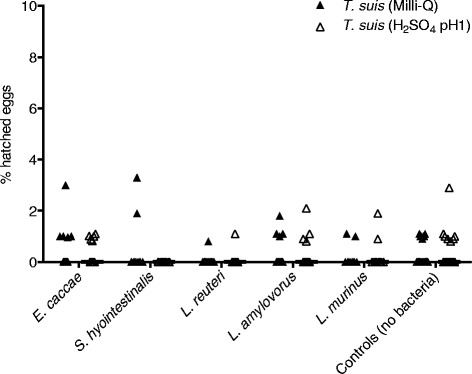
*Trichuris suis* eggs incubated with Gram-positive bacteria in RPMI-1640 without antimicrobials. Samples were incubated for 20 hours at 37 °C. Individual counts of *T. suis* (Milli-Q or H_2_SO_4_ pH1) and median for all experiments with *Enterococcus caccae* (n = 20), *Streptococcus hyointestinalis* (n = 10), *Lactobacillus reuteri* with *T. suis* in Milli-Q (n = 15), *L. reuteri* with *T. suis* in H_2_SO_4_ pH1 (n = 20)*, L. amylovorus* (n = 15), and *L. murinus* (n = 10). Controls represent *T. suis* eggs without the addition of bacteria (n = 25)

None of the tested bacteria induced significant hatching of *T. suis* eggs after 4 (average hatching less than 0.4 %) or 20 h post-incubation (Fig. 3).

**Fig. 4:**
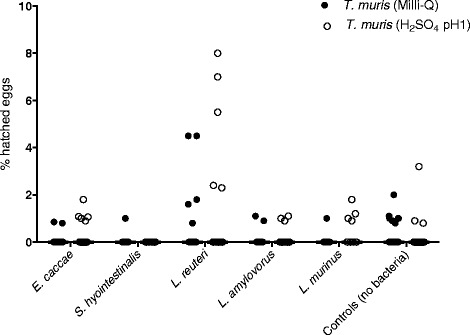
*Trichuris muris* eggs incubated with Gram-positive bacteria in RPMI-1640 with antimicrobials. Samples were incubated for 20 hours at 37 °C. Individual counts of *T. muris* (Milli-Q or H_2_SO_4_ pH1) and median for all experiments with *Enterococcus caccae* (n = 20), *Streptococcus hyointestinalis* (n = 10), *Lactobacillus reuteri*with *T. muris* in Milli-Q (n = 20), *Lactobacillus reuteri* with *T. muris* in H_2_SO_4_ pH1 (n = 15), *L. murinus* (n = 10), and *L. amylovorus* (n = 15). Controls represent *T. muris* eggs without the addition of bacteria (n = 25)

Samples incubated with antimicrobials showed consistently low egg hatching of *T. muris* (Fig. 4) and *T. suis* eggs (average hatching was less than 0.6 %) after 20 h incubation. In all experiments, the control group with whipworm eggs without the addition of bacteria had low hatching (average hatching less than 0.5 %).

## Discussion

The use of individual bacterial species [16–18] or contents from the caecum [18, 21, 22] and the upper colon [22] in hatching *T. muris* eggs, points to close interactions between commensal bacteria and parasites within their niche. Such interactions may also explain observations of parasite’s ability to modulate the intestinal microbiota [23–27], as shown by an increase of lactobacilli during chronic *T. muris* infection [26]. In the large intestine of Göttingen minipigs and mice, microbiota is primarily composed of the phyla Firmicutes and Bacteroidetes [28–30], while Proteobacteria comprises 0.6 % in Göttingen minipigs [28].

In the present study, *T. muris* eggs hatched with *E. coli* (Proteobacteria), *E. caccae* (Firmicutes), *S. hyointestinalis* (Firmicutes), *L. reuteri*, and *L. amylovorus* (Firmicutes), while *T. suis* had overall very low hatching. However, *T. muris* egg hatching was inconsistent and highly variable with the Gram-positive bacteria.

Due to dietary differences between a mouse and a pig, host-specific bacterial composition may account for the observed discrepancy in the hatching of eggs of the two whipworm species. Although only a few bacteria were presently selected for comparison, selected bacterial species belong to the genera associated with pig and mouse intestinal tract.

Different bacteria may have different mechanisms in which they trigger eggs to hatch. Hayes et al. reported that *T. muris* eggs hatched through close physical contact with *E. coli*, and suggested that other mechanisms may exist for *Pseudomonas aeruginosa* and *Staphylococcus aureus* [18]. Since the large intestine contains a diverse microbial community (mostly anaerobes) [31], it is possible that a combination of different bacteria with their specific mechanisms, rather than one specific bacterium, is needed to hatch *T. suis* eggs. Whether a specific bacteria-induced hatching pattern is characteristic of other whipworms eggs (e.g. *T. trichiura* and *T. vulpis*) is still undefined. While Areekul et al. reported *T. trichiura* and *T. vulpis* co-infections in stools of 10.7 % *Trichuris* positive children in Thailand [32], other reported human cases of *T. vulpis* infection are brought into question (reviewed in [33]).

On the other hand, the similarities between pig and human gut microbiota (reviewed in [34]) and positive treatment effect of *T. suis* eggs in patients with immune-mediated diseases [7–10], indicate that *T. suis* eggs hatch in the human gut. In addition, pigs have been experimentally cross-infected with *T. trichiura*, although the development did not reach a patent infection [35]. Therefore, it can be speculated that a common bacterial stimulus exists between *T. suis* and *T. trichiura*. Recently, a mix of *T. trichiura* and *T. suis* sequence types was identified in three human-originating worms [36]. Even though a few studies either observed the presence of a single male adult worm in the human gut [37] or low grade *T. suis* egg excretion under experimental infections [35, 38], humans are not considered either natural or suited host for *T. suis*. Furthermore, during repeated dosing in clinical studies, *T. suis* eggs are absent in the stool samples [8, 9], which underline that patent *T. suis* infection is limited to pigs, where the full life cycle is confined.

Although the present study tested a limited number of bacteria, our findings together with previous limited observations on *Trichuris* cross-species transmission, suggests that *Trichuris* has a narrower host-specificity window compared to other nematodes, such as *Ascaris* [39].

To further study the parasite-bacteria interaction and host-parasite specificity, *T. suis* and *T. muris* eggs may be cross-incubated with mucosal scrapings from the intestine of a mouse and pig, respectively. Inclusion of other whipworm species in such experiment, e.g. human *T. trichiura* and canine *T. vulpis*, may provide further insight into the specific parasite adaptation to the host gut environment.

## Conclusions

The present study is the first to compare eggs of two whipworm species in relation to bacterial-dependent hatching. Although *T. muris* successfully hatched in the presence of the different *E. coli* strains, the egg hatching showed high variability in the presence of five tested Gram-positive bacteria after overnight incubation without the addition antimicrobials. In contrast, none of the tested bacteria triggered hatching of *T. suis* eggs. This suggests that the co-evolution of the parasite and host, and the adaptation to host’s microbiota has resulted in unique host-specific hatching stimuli of *T. muris* and *T. suis* eggs.
